# Management options for large plants of glyphosate-resistant feather fingergrass (*Chloris virgata*) in Australian fallow conditions

**DOI:** 10.1371/journal.pone.0261788

**Published:** 2021-12-23

**Authors:** Bhagirath Singh Chauhan, Mark Congreve, Gulshan Mahajan

**Affiliations:** 1 The Centre for Crop Science, Queensland Alliance for Agriculture and Food Innovation (QAAFI), The University of Queensland, Gatton, Queensland, Australia; 2 School of Agriculture and Food Sciences (SAFS), The University of Queensland, Gatton, Queensland, Australia; 3 Department of Agronomy, Chaudhary Charan Singh Haryana Agricultural University, Hisar, Haryana, India; 4 Independent Consultants Australia Network (ICAN), Reedy Creek, Queensland, Australia; 5 Punjab Agricultural University, Ludhiana, Punjab, India; Canakkale Onsekiz Mart University, TURKEY

## Abstract

*Chloris virgata* has become one of the most difficult glyphosate-resistant (GR) grass weeds in summer fallows in the eastern region of Australia. It germinates in several cohorts following rainfall events; therefore, growers are often tempted to wait for most of the weeds to emerge before herbicide application. However, by that time, some seedlings have reached an advanced stage and there is limited information on the efficacy and reliability of alternate herbicides when targeting large plants of GR *C*. *virgata*. A series of experiments were conducted to determine the efficacy of alternate herbicides for the control of GR *C*. *virgata*. Haloxyfop (80 g a.i. ha^-1^) on its own, in mixtures, or sequential applications of haloxyfop and paraquat or glufosinate provided 97 to 100% mortality of the 8–10 leaf stage plants. Glufosinate (1500 g a.i. ha^-1^) also provided complete control of plants at this growth stage. For larger plants at the 24–28 leaf stage, glufosinate, with or without additional tank-mixed adjuvants, generally did not provide full control, however did show very high levels of biomass reduction and panicle suppression at application rates of 750 or 1500 g a.i. ha^-1^. Haloxyfop (40 to 160 g a.i. ha^-1^) and clethodim (180 g a.i. ha^-1^) on their own achieved 96 to 100% mortality at this growth stage. When applied to large plants (40–50 leaf stage), a tank-mix of isoxaflutole plus paraquat demonstrated significantly higher levels of plant mortality and biomass reduction than either herbicide used alone, and this mixture appears to be synergistic when tested via the Colby equation for synergy or antagonism. Plant mortality was greater (83%) when isoxaflutole (75 g a.i. ha^-1^) plus paraquat (300 g a.i. ha^-1^) was taken up through the foliage and soil, compared with the foliage alone. This study identified alternative herbicide options for large plants of GR *C*. *virgata*.

## Introduction

Feather fingergrass (*Chloris virgata* Sw.), commonly known as feathertop Rhodes grass in Australia, has become a problematic grass weed in the eastern states of Australia. Nationally, it is ranked in the top eight grain-crop weeds in terms of its impact on yield and revenue losses [1]. It is estimated to cost AUD 7.7 million per annum to Australian grain growers, in addition to causing revenue losses and additional expense to cotton growers and councils. This weed was reported to infest more than 100,000 ha of cropping land in 2016 [1]. Since then, the infestation has continued to increase. In a survey conducted in 2010 across Queensland and New South Wales, Australia, *C*. *virgata* was ranked in the top 20 most common weeds in cotton cropping systems [2]. Another similar survey undertaken 5 years later ranked it as the sixth most common weed species in cotton cropping systems [3].

*Chloris virgata* possesses a C_4_ photosynthetic pathway and can produce more than 140,000 seeds per plant in fallow conditions [4]. The seeds of this species are small and lightweight, which easily disperse via wind or overland water flow and sometimes via livestock. Being a small-seeded species with great potential for emergence from the topsoil layers, *C*. *virgata* has become a dominant weed species in conservation agriculture systems in eastern Australia [5]. Due to over-reliance on glyphosate for control in fallows in conservation agriculture systems, several biotypes of this weed have developed resistance to this herbicide [6]. A recent study showed target-site EPSPS mutations confer resistance to glyphosate in *C*. *virgata* [6]. There is a need to evaluate alternative herbicide options for the control of *C*. *virgata* in fallow situations.

Tank mix and sequential applications of herbicides are effective in controlling herbicide-resistant and hard to control weeds, including *C*. *virgata* [5, 6]. In a recent study, for example, four populations of glyphosate-resistant (GR) *C*. *virgata* were tested against sequential treatments of glyphosate or glufosinate followed by paraquat [6]. These treatments provided complete control of the weed. However, the herbicides were applied at the 4–5 leaf stage of the species. Herbicides are usually recommended at a young seedling stage for maximum consistency of results, but often due to environmental constraints growers may end up spraying herbicides on large plants, especially during the fallow phase. Historically many growers wait for a greater number of weeds to emerge to save costs on herbicides and fuel; however, by that time, some weeds may have passed the optimum spray stage. Therefore, alternative herbicides need to be evaluated at advanced growth stages of *C*. *virgata*.

Herbicide efficacy may be increased by using adjuvants in certain situations. For example, mixing several adjuvants with glyphosate and glufosinate was found to increase velvetleaf (*Abutilon theophrasti* Medik.) control [7]. Adjuvants may modify spray retention on the leaf surface, coverage, and deposit formation [8]. Information is not readily available on the efficacy of herbicides mixed with adjuvants on *C*. *virgata*.

In Australia, isoxaflutole is a soil-applied isoxazole herbicide that controls susceptible species by inhibiting 4-hydroxyphenyl-pyruvate-dioxygenase (4-HPPD) [9]. When applied to the soil, isoxaflutole and its herbicidally active metabolite diketonitrile, are taken up predominantly through the roots. Although isoxaflutole is typically recommended as a PRE herbicide in Australia, the product label recommends mixing with a knockdown herbicide such as paraquat if weeds have already germinated in a fallow situation. In a recent study, a tank mix treatment of isoxaflutole (75 g a.i. ha^-1^) plus paraquat (500 g a.i. ha^-1^) provided 92 to 99% control of flowering *C*. *virgata* plants [5]. In this situation, it is believed that the mixture of these two herbicides was absorbed mainly through the foliage, as plants were quite large at spraying. Therefore, there is a desire to understand the absorption of the mixture of isoxaflutole and paraquat, especially in the situation where most of the herbicide solution deposits on the foliage of larger plants. Information is also needed to evaluate if the mixture of the two herbicides increases efficacy on large plants of *C*. *virgata* compared with the sole application of paraquat. Therefore, a series of pot experiments were conducted to evaluate the performance of alternative herbicides options for the control of GR *C*. *virgata*.

## Materials and methods

Seeds of *C*. *virgata* were collected from at least 50 plants in April 2017 from a sorghum field in Cecil Plains (-27.2935, 151.1283), Queensland, Australia. The authors confirm that the owner of the land gave permission to collect the weed seeds, as well as that the field studies did not involve endangered or protected species. Details of this population are given in Desai et al. [6]. The seeds were planted in pots (25 cm diameter) and grown for bulk seed production in 2019 at the Gatton Research Farm (-27.5350, 152.3376) of the University of Queensland, Queensland. Seeds collected from these plants were stored in a laboratory in plastic containers at room temperature (25 ± 2 C) until used in the experiments. As the first and the last authors work in this institute, no permission was needed to conduct the work.

Experiments were conducted in two runs: August to December 2020 (first run) and January to April 2021 (second run). Herbicide treatments were applied using a research track sprayer that delivered 108 L ha^-1^ spray volume through flat fan nozzles (TeeJet XR 110015) at a spray pressure of 200 kPa. Pots were filled with potting mix (pH 5.6 and electrical conductivity 1.6 dS m^-1^; Centenary Landscape, Australia). Pots were placed on benches in an open, outdoor area and irrigated regularly (3–4 times each day) using an automated sprinkler system. No water was given for 24 h after spray. After 24 h of spray, pots were sub-irrigated for 3 days. Overhead watering was recommenced 3 days after spraying.

### Experiment 1. Adjuvants with glyphosate

This experiment was designed to evaluate if the addition of tank-mixed adjuvants could improve the effectiveness of glyphosate when applied to large GR *C*. *virgata* plants. A single rate of glyphosate was applied, with or without a range of commonly used tank-mixed adjuvants. Pots (20 cm diameter) were filled with potting mix and 4–5 seeds of *C*. *virgata* were planted in the center of the pot. Only one plant per pot was maintained after emergence. Herbicide treatments (Table 1) were applied at the 40–50 leaf stage. There was also a nontreated control treatment. There were six replicates in each experimental run.

**Table 1:** Leaf stage and herbicide treatments included in different experiments.

Experiment 1	Experiment 2	Experiment 3	Experiment 4	Experiment 5	Experiment 6
Leaf stage					
40–50 leaves/plant	8–10 leaves/plant	Small: 8–10 leaves/plant	24–28 leaves/plant	40–50 leaves/plant	40–50 leaves/plant
		Large: 24–28 leaves/plant			
Herbicide treatments (g a.e. or a.i./ha)					
Control	Control	Control	Control	Control	Control
Glyphosate 740	Glyphosate 740	Glufosinate 375	Clethodim 45	Paraquat 150	Paraquat 300
Glyphosate 740 + 2% AMS	Glyphosate 740 + 2,4-D 700	Glufosinate 375 + 2% AMS	Clethodim 90	Paraquat 300	Isoxaflutole 37.5
Glyphosate 740 + 2% urea	Glyphosate 740 fb paraquat 600	Glufosinate 375 + 0.5% BS1000	Clethodim 180	Paraquat 600	Isoxaflutole 75.0
Glyphosate 740 + 0.5% BS1000	Glyphosate 740 + 2,4-D 700 fb paraquat 600	Glufosinate 375 + 0.5% Hasten	Haloxyfop 40	Paraquat 900	Isoxaflutole 37.5 + paraquat 300
Glyphosate 740 + 0.5% Hasten	Glufosinate 750	Glufosinate 750	Haloxyfop 80	Isoxaflutole 18.75	Isoxaflutole 75.0 + paraquat 300
	Glyphosate 740 fb glufosinate 750	Glufosinate 750 + 2% AMS	Haloxyfop 160	Isoxaflutole 37.5	
	Glyphosate 740 + 2,4-D 700 fb glufosinate 750	Glufosinate 750 + 0.5% BS1000	Clethodim 90 + haloxyfop 80	Isoxaflutole 75.0	
	Haloxyfop 80	Glufosinate 750 + 0.5% Hasten		Isoxaflutole 112.5	
	Haloxyfop 80 fb paraquat 600	Glufosinate 1500		Isoxaflutole 18.75 + paraquat 300	
	Haloxyfop 80+ paraquat 600	Glufosinate 1500 + 2% AMS		Isoxaflutole 37.5 + paraquat 300	
	Haloxyfop 80 fb glufosinate 750	Glufosinate 1500 + 0.5% BS1000		Isoxaflutole 75.0 + paraquat 300	
	Haloxyfop 80 + glufosinate 750	Glufosinate 1500 + 0.5% Hasten		Isoxaflutole 18.75 + paraquat 600	
	Paraquat 600			Isoxaflutole 37.5 + paraquat 600	
				Isoxaflutole 75.0 + paraquat 600	

Abbreviations: AMS, ammonium sulfate; fb, followed by.

In experiments 2, 4 and 5, BS1000 (1%) was added with paraquat treatments; Supercharge (1%) with clethodim; Hasten (1%) with haloxyfop.

### Experiment 2. Tank mix and sequential herbicide applications

Applying herbicide applications in mixtures, or in sequence (commonly known as the ‘double-knock technique’ in Australia) often improves weed control. Sequential applications are often favored especially where both systemic and contact herbicides are required, with normally the systemic herbicide being applied first and time allowed for translocation, before the contact herbicide is applied. This experiment evaluated the efficacy of glyphosate (740 g a.e. ha^-1^) +/- 2,4-D (700 g a.e. ha^-1^), glufosinate (750 g a.i. ha^-1^), haloxyfop (80 g a.i. ha^-1^), and paraquat (600 g a.i. ha^-1^) when applied (at standard rates) either alone or as the first pass in a sequential program; with paraquat or glufosinate applied in treatments where a second sequential application was required. Pots (20 cm diameter) were filled with potting mix and 12 seeds of *C*. *virgata* were planted uniformly in the pots. After emergence, plants were thinned to maintain 5 plants per pot. Herbicide treatments (Table 1) were applied at the 8–10 leaf stage. For sequential herbicide treatments, the second herbicide was applied 7 d after the first herbicide application. There was a nontreated control treatment with three replications in each experimental run.

### Experiment 3. Leaf stage and glufosinate-based treatments

Glufosinate can be used to control a range of weeds in chemical fallows in Australia. Anecdotal evidence suggests that glufosinate may also be effective against *C*. *virgata*, so an experiment was designed to test different application rates (0.5x, 1x, and 2x of the recommended rate; 1x = 750 g a.i. ha^-1^) weed size, and tank-mixed adjuvants. *Chloris virgata* seeds (12) were planted in plastic pots (20 cm diameter) filled with potting mix. Only 5 plants per pot were maintained after emergence. Herbicide treatments (Table 1) were applied at two growth stages: 8–10 and 24–28 leaves per plant. Glufosinate was used at three different doses with different adjuvants added as a tank-mix before application. There was a nontreated control treatment and there were three replications in each experimental run.

### Experiment 4. Clethodim and haloxyfop treatments

Australian product labels of certain clethodim and haloxyfop formulations recommend use for control of *C*. *virgata* in certain use situations; however, application is usually recommended to young/small weeds (90 g a.i. ha^-1^ for clethodim and 80 g a.i. ha^-1^ for haloxyfop). This experiment evaluated performance at a range of application rates (0.5x to 2x the recommended rates) when applied to advanced *C*. *virgata*. As described above, 5 plants per pot were maintained after emergence. Herbicides (Table 1) were applied at the 24–28 leaf stage. A nontreated control treatment was included and each experimental run had three replications.

### Experiment 5. Isoxaflutole and paraquat mixtures

As previously mentioned, there is evidence that mixtures of isoxaflutole and paraquat may improve control of *C*. *virgata* when applied as a foliar application to existing plants. In this experiment, the response of *C*. *virgata* plants to different doses (0.5x, 1x, 2x, and 4x; 1x of isoxaflutole = 37.5 g a.i. ha^-1^) of isoxaflutole and paraquat (0.25x, 0.5x, 1x, and 1.5x; 1x of paraquat = 600 g a.i. ha^-1^) either applied alone or in mixture was studied. Potting mix was filled in 12 cm diameter plastic pots and 4–5 seeds of *C*. *virgata* were planted in the center of each pot. Only one plant per pot was maintained after emergence. Plants were allowed to grow as large as possible but were not allowed to produce inflorescences. Herbicide treatments (Table 1) were applied at the 40–50 leaf stage (approximately 30 cm in height). A non-treated control treatment was included, and each experimental run had six replications.

### Experiment 6. Isoxaflutole and paraquat applications to roots and foliage

In this experiment, we sought to evaluate the efficacy from isoxaflutole (37.5 or 75 g a.i. ha^-1^) or isoxaflutole (37.5 or 75 g a.i. ha^-1^) + paraquat (300 g a.i. ha^-1^) when applied to either roots and foliage, or foliage only. Seeds (4–5) of *C*. *virgata* were planted in 12 cm diameter plastic pots and only one plant per pot was maintained after emergence. Herbicide treatments (Table 1) were applied at the 40–50 leaf stage and a nontreated control treatment was also included. Plants were allowed to grow until they covered at least 50% of the pot area exposed to the spray to allow soil uptake. To create different herbicide uptake treatments, where required a shroud made of cardboard was placed around the plants to prevent excess spraying from getting to the soil surface during application. The cardboard was left in place for 3 days after herbicide application. Each experimental run had six replications (i.e., 6 pots x 2 runs).

### Statistical analysis

Each experiment was conducted in a randomized complete block design. All treatments within a replication were randomized on benches and pots were randomly rotated every week to avoid the edge effect. Data was taken 28 d after herbicide spray in each experiment. Surviving plants (defined as at least one new leaf following herbicide application) were counted and cut at the pot level to measure above-ground plant biomass. Plant samples were placed in an oven at 70°C for at least 72 h and weighed to measure their dry biomass. Live panicle numbers per pot were also counted.

Where the analysis of variance (ANOVA) showed that there was no difference between the runs and the interaction between the experimental runs and treatments was also nonsignificant [10], then the data were pooled over the experimental runs. Data were square-root transformed [√(x +0.5)], where needed, to improve the homogeneity of variance. Means were separated using LSD at the 5% level of significance.

Experiment 5 was designed to allow testing of synergy of isoxaflutole and paraquat mixtures using the Colby equation E = X + Y–X*Y/100 where E = expected result for the mixture; X = actual result from product A applied alone, and Y = actual result from product B applied alone [11]. If the actual result from the mixture is greater than the expected result from the Colby equation then the mixture is deemed to be synergistic.

## Results and discussion

### Experiment 1. Adjuvant with glyphosate

The addition of adjuvants with glyphosate (740 g a.e. ha^-1^) did not improve the control of *C*. *virgata* compared with nontreated control plants. Irrespective of treatment, 100% of plants survived and dry matter ranged from 2.71 to 3.65 g plant^-1^. *C*. *virgata* produced 9.2 to 11.8 panicles plant^-1^. These results suggest that this population has a level of glyphosate resistance which is expressed in plants of this size that was able to overcome the application rate of glyphosate chosen. The addition of ammonium sulfate (AMS), urea, or commercial surfactants did not provide any additional advantage over glyphosate alone for the control of this population of GR *C*. *virgata*. Our results suggest that adjuvants with glyphosate do not improve efficacy. In previous studies, the addition of AMS to glyphosate has been found to improve glyphosate uptake through plant foliage [12]. In another study, the control of Johnsongrass [*Sorghum halepense* (L) Pers.] with glyphosate was found to be increased with the addition of AMS [13].

### Experiment 2. Tank mix and sequential herbicide application

Although *C*. *virgata* plants in this trial were younger (8–10 leaves plant^-1^) than in the previous trial, glyphosate alone, or in a mix with 2,4-D (700 g a.e. ha^-1^), still did not cause mortality when applied as a single application at the selected application rate of 740 g a.e. ha^-1^ (Table 2). Table 2 also shows that, as a single application, glufosinate provided 97% and haloxyfop 100% control of *C*. *virgata* at the application rates applied. Currently, there are no claims for control of *C*. *virgata* on Australian glufosinate product labels; however, the application rate used in this experiment is a standard rate (750 g a.i. ha^-1^) used to control several other weed species.

**Table 2:** Effect of herbicide mixtures and sequential herbicides on survival, panicle production, and shoot biomass of glyphosate-resistant *Chloris virgata*. Herbicides were sprayed at the 8–10 leaf stage.

Treatment	Survival	Panicle	Biomass
	%	number pot^-1^	g pot^-1^
Control	100	17.7	9.32
Glyphosate 740	100	14.0	8.45
Glyphosate 740 + 2,4-D 700	100	13.5	9.73
Glyphosate 740 fb paraquat 600	67	1.8	1.64
Glyphosate 740 + 2,4-D 700 fb paraquat 600	77	3.0	3.86
Glufosinate 750	3	0.2	0.11
Glyphosate 740 fb glufosinate 750	20	0.3	0.47
Glyphosate 740 + 2,4-D 700 fb glufosinate 750	34	0.8	0.69
Haloxyfop 80	0	0.0	0.00
Haloxyfop 80 fb paraquat 600	0	0.0	0.00
Haloxyfop 80 + paraquat 600	3	0.0	0.01
Haloxyfop 80 fb glufosinate 750	0	0.0	0.00
Haloxyfop 80 + glufosinate 750	0	0.0	0.00
Paraquat 600	43	2.2	2.77
LSD	28	2.9	2.46

Abbreviation: Fb, followed by.

BS1000 (1%) was added with paraquat; Hasten (1%) with haloxyfop.

Typically it is not usually recommended to tank-mix fast-acting contact herbicides with herbicides that require time to translocate throughout the plant. In this trial, tank mixing the contact herbicide glufosinate with the systemic haloxyfop still achieved 100% control as a single application; while a tank mix of haloxyfop with the other contract herbicide paraquat resulted in a non-significant reduction in control to 97%. This maintenance of control with these tank mixes may be as a result of the high level of efficacy achieved by haloxyfop alone, and may not be repeatable at lower application rates, or under less favorable application conditions.

Where sequential application of either paraquat (600 g a.i. ha^-1^) or glufosinate (750 g a.i. ha^-1^) followed glyphosate (740 g a.e. ha^-1^) or glyphosate (740 g a.e. ha^-1^) + 2,4-D (700 g a.e. ha^-1^), there was an increase in phytotoxicity, measured as either improved mortality or reduced panicle count and biomass, when compared to the glyphosate alone treatments. However, there was generally no significant improvement from these sequential applications compared to the respective paraquat or glufosinate treatments applied alone, implying that paraquat or glufosinate were the most significant contribution to the result achieved. Where haloxyfop (80 g a.i. ha^-1^) was applied alone or with a sequential application of either paraquat (600 g a.i. ha^-1^) or glufosinate (750 g a.i. ha^-1^), all treatments provided 100% mortality so it is difficult to make any additional comments.

Australian growers, in general, often desire to mix glyphosate and 2,4-D to achieve broad-spectrum weed control in chemical fallows. The results from this experiment suggest that adding 2,4-D with glyphosate may further compromise the efficacy of glyphosate, especially on large GR *C*. *virgata* plants, although the reduction in performance in this experiment was not statistically significant. A separate study confirmed an antagonism effect of glyphosate and 2,4-D mixtures in glyphosate susceptible, as well as GR populations of *C*. *virgata* (Mahajan and Chauhan, unpublished data). However, the glyphosate dose used in that study was four times the glyphosate dose used in this experiment.

Sequential herbicide applications have been found to be very effective for controlling difficult to kill and, in certain situations for GR grass weed biotypes [6, 14]. The majority of previous studies have evaluated the performance of the sequential application of glyphosate followed by paraquat, whereas in this study we have shown that glufosinate may be an alternate sequential option for GR *C*. *virgata* management.

Glufosinate product labels state that warm (but not hot) temperature; high humidity and good application coverage are essential for reliable performance. These conditions may often be difficult to achieve in Australian commercial use situations (low humidity and high heat) where *C*. *virgata* is regularly a problem. However, the performance of glufosinate under the controlled conditions and soil moisture status where this experiment was conducted suggests that further research should be undertaken to confirm the reliability of glufosinate against this problem weed, where new herbicide control strategies are required.

### Experiment 3. Leaf stage and glufosinate-based treatments

In Experiment 2, glufosinate provided effective control of small (8–10 leaves plant^-1^) GR *C*. *virgata*. Therefore, in this experiment, the performance of different doses of glufosinate (with and without adjuvants) was evaluated on both small and large plants (24–28 leaves plant^-1^). Three application rates were tested– 750 g a.i. ha^-1^ being a common use rate for several other weed species in Australian chemical fallow situations, plus ½ and 2x this rate.

Compared with the nontreated control treatment, all glufosinate-based treatments (label rate: 750 g a.i. ha^-1^) provided a level of control of small plants of *C*. *virgata* (Table 3). Glufosinate at a suboptimal dose (375 g a.i. ha^-1^) provided only 60% mortality, which increased to 93% at 750 g a.i. ha^-1^ and 100% at 1500 g a.i. ha^-1^. The addition of any of the three adjuvants tested did not provide any additional statistically significant advantage over glufosinate alone when sprayed at the 8–10 leaf stage. When results from this experiment are viewed in association with results from Experiment 2 it suggests that the application rate of 750 g a.i. ha^-1^ is likely to provide useful, but possibly not complete, control of *C*. *virgata* when targeting 8–10 leaf plants, and higher application rates may be beneficial.

**Table 3:** Effect of different doses of glufosinate (with and without adjuvants) on survival, panicle production, and shoot biomass of glyphosate-resistant *Chloris virgata*. Herbicides were sprayed on small (8–10 leaf stage) and large (24–28 leaf stage) plants.

Treatment	Survival		Panicle		Biomass	
	Small	Large	Small	Large	Small	Large
	%		number pot^-1^		g pot^-1^	
Control	100	100	1.7 (3.3)	2.4 (6.0)	3.0 (9.30)	3.5 (12.60)
Glufosinate 375	40	80	0.7 (0.0)	0.7 (0.0)	0.8 (0.13)	1.6 (2.06)
Glufosinate 375 + 2% AMS	20	73	0.7 (0.0)	0.7 (0.0)	0.8 (0.08)	1.2 (1.04)
Glufosinate 375 + 0.5% BS1000	40	97	0.7 (0.0)	1.1 (0.8)	0.8 (0.13)	1.7 (2.45)
Glufosinate 375 + 0.5% Hasten	37	87	0.7 (0.0)	1.3 (1.3)	0.8 (0.07)	1.6 (2.46)
Glufosinate 750	7	43	0.7 (0.0)	0.7 (0.0)	0.7 (0.01)	0.9 (0.41)
Glufosinate 750 + 2% AMS	13	43	0.7 (0.0)	0.7 (0.0)	0.8 (0.21)	0.9 (0.36)
Glufosinate 750 + 0.5% BS1000	3	63	0.7 (0.0)	0.7 (0.0)	0.8 (0.11)	1.3 (1.26)
Glufosinate 750 + 0.5% Hasten	7	40	0.7 (0.0)	0.7 (0.0)	0.7 (0.01)	0.9 (0.36)
Glufosinate 1500	0	27	0.7 (0.0)	0.7 (0.0)	0.7 (0.00)	0.8 (0.16)
Glufosinate 1500 + 2% AMS	0	13	0.7 (0.0)	0.7 (0.0)	0.7 (0.00)	0.7 (0.06)
Glufosinate 1500 + 0.5% BS1000	0	30	0.7 (0.0)	0.7 (0.0)	0.7 (0.00)	0.8 (0.12)
Glufosinate 1500 + 0.5% Hasten	0	0	0.7 (0.0)	0.7 (0.0)	0.7 (0.00)	0.7 (0.00)
LSD	23		0.3		0.4	

Abbreviations: AMS, ammonium sulfate.

Panicle and biomass data were square transformed before analysis. Original values are given in parentheses.

For larger plants (24–28 leaves plant^-1^), a strong dose-response was evident; however, all rates failed to achieve what would be considered a ‘commercially acceptable’ level of plant mortality. The addition of tank-mixed adjuvants generally did not result in consistent or statistically significant improvement in the level of control on these large plants. Glufosinate plus the addition of Hasten^®^ (a blend of esterified vegetable oil and nonionic surfactants) did result in a statistically valid improvement in efficacy when applied at the highest application rate (1500 g a.i. ha^-1^) only, and this was the only treatment to achieve 100% control. The addition of Hasten to lower rates of glufosinate did not improve control.

Compared with the nontreated control treatment, all glufosinate-based treatments reduced panicle production by *C*. *virgata* (Table 3). None of the herbicide-treated small plants produced panicles, but some large plants treated with glufosinate at the suboptimal dose (375 g a.i. ha^-1^) produced 0.8 to 1.3 panicles plant^-1^. Similarly, biomass production was significantly reduced in all glufosinate-treated plants compared with the control plants (Table 3). Irrespective of growth stage and herbicide treatment, a greater than 80% reduction in biomass was achieved compared with the control.

In previous studies, AMS has been found to increase the efficacy of glufosinate on weeds through increasing foliar absorption [15]. However, in the current study, AMS did not improve the efficacy of glufosinate on *C*. *virgata*. Only one surfactant (Hasten^®^) improved the efficacy of large plants of *C*. *virgata*, but only at the highest dose rate tested. Interactions between surfactants, herbicide formulations, environmental conditions, and spray water quality are complex. Under these trial conditions, and with this particular glufosinate formulation, we were not able to show consistent benefit from the addition of a selected range of tank-mixed adjuvants.

### Experiment 4. Clethodim and haloxyfop treatments

This experiment was conducted to evaluate the performance of Group 1 (acetyl co-enzyme A carboxylase inhibitor) herbicides against large (24–28 leaves plant^-1^) *C*. *virgata*. In Australia, clethodim product labels recommend control of 5-leaf to fully tillered *C*. *virgata* in a range of summer crops when applied at rates of 90 to 120 g a.i. ha^-1^ (the lower dose will provide effective control if applied under ideal conditions to weeds that are smaller, actively growing and free from temperature or water stress). In this experiment clethodim at 90 g a.i. ha^-1^, and half that rate, failed to provide 100% mortality of these large plants, although panicle production and biomass were severely curtailed (Table 4). At the high application rate, which is above existing label recommendations (90 g a.i. ha^-1^), 97% plant mortality was achieved. It should be noted that this experiment was conducted in the absence of crop competition that would normally be expected to assist herbicide performance when applied per the labeled use pattern. This dose-response experiment largely supports the current clethodim label directions for control of *C*. *virgata*.

**Table 4:** Effect of different doses of clethodim and haloxyfop on survival, panicle production, and shoot biomass of glyphosate-resistant *Chloris virgata*. Herbicides were sprayed at the 24–28 leaf stage.

Treatment	Survival	Panicle	Biomass
	%	number pot^-1^	g pot^-1^
Control	100	18.2	10.22
Clethodim 45	20	0	0.07
Clethodim 90	29	0	0.02
Clethodim 180	4	0	0.01
Haloxyfop 40	0	0	0
Haloxyfop 80	0	0	0
Haloxyfop 160	0	0	0
Clethodim 90 + haloxyfop 80	0	0	0
LSD	13	1.7	0.58

Supercharge (1%) was added with clethodim and Hasten (1%) with haloxyfop.

Haloxyfop is recommended to control 2-leaf to early tillering *C*. *virgata* in chemical fallows at 80 g ha^-1^ on some, but not all, Australian product labels. Efficacy of haloxyfop in this experiment was exceptional, with 100% plant mortality achieved from all application rates tested (40 to 160 g a.i. ha^-1^) (Table 4). A treatment of clethodim at 90 g a.i. ha^-1^ plus haloxyfop at 80 g ha^-1^ also achieved 100% control. These results support other similar research such as a recent study conducted in New South Wales that reported effective control (96–98%) of *C*. *virgata* when haloxyfop at 156 g a.i. ha^-1^ was applied on flowering plants [5]. Similar to our results, a recent report suggested that the ‘fop’ herbicides (propaquizafop and haloxyfop) may perform better on *C*. *virgata* than ‘dim’ herbicides (butroxydim and clethodim) when used as stand-alone applications [16].

It should be emphasized that control of *C*. *virgata* with Group 1 herbicides in chemical fallow situations is heavily dependent on achieving excellent application coverage and plants that are actively growing. This is much easier to achieve in pot experiments than under commercial application conditions–for example, plants in this experiment were watered daily, which is unlikely to occur in commercial situations. Typically, these Group 1 herbicides do not perform consistently on large grass weeds under a range of commercial field conditions, so herbicide registrants typically take a more conservative approach to weed size and application rate recommendations to ensure reliable performance.

Additionally, Group 1 herbicides are noted to be prone to selection of resistant biotypes when used frequently on the same field. For this reason, it is a standard recommendation in Australia when applying these herbicides to chemical fallow (with an absence of crop competition) to target small weeds with robust application rates to ensure high levels of control, and always follow with a sequential application of a contact herbicide, such as paraquat.

### Experiment 5. Isoxaflutole and paraquat mixtures

In the dose-response experiment, paraquat ranging from 150 to 900 g a.i. ha^-1^ did not control large plants (40–50 leaves plant^-1^) of *C*. *virgata* (Table 5). Similarly, isoxaflutole ranging from 18.8 to 112.5 g a.i. ha^-1^ resulted in 0 to 25% plant mortality.

**Table 5:** Effect of different doses of paraquat, isoxaflutole and their mixtures on survival, panicle production, and shoot biomass of glyphosate-resistant *Chloris virgata*. Percent biomass reduction and expected Colby equation values from mixtures of isoxaflutole and paraquat were also calculated. Herbicides were sprayed at the 40–50 leaf stage.

Treatment	Survival	Panicle	Biomass	Biomass reduction versus untreated	Expected valued (Colby equation)
	%	number pot^-1^	g pot^-1^	%	%
Control	100	6.4	3.85	-	-
Paraquat 150	100	5.4	2.40	37.7	-
Paraquat 300	100	4.8	1.93	49.9	-
Paraquat 600	100	1.8	1.20	68.8	-
Paraquat 900	92	1.8	1.36	64.7	-
Isoxaflutole 18.75	100	4.1	3.06	20.5	-
Isoxaflutole 37.5	83	4.3	1.80	53.2	-
Isoxaflutole 75.0	83	2.8	1.65	57.1	-
Isoxaflutole 112.5	75	1.3	0.72	81.3	-
Isoxaflutole 18.75 + paraquat 300	75	1.7	0.56	85.5	60.2
Isoxaflutole 37.5 + paraquat 300	83	0.1	0.17	95.6	76.6
Isoxaflutole 75.0 + paraquat 300	17	0.0	0.01	99.7	78.5
Isoxaflutole 18.75 + paraquat 600	92	0.4	0.23	94.0	75.2
Isoxaflutole 37.5 + paraquat 600	33	0.0	0.10	97.4	85.4
Isoxaflutole 75.0 + paraquat 600	8	0.0	0.01	99.7	86.6
LSD	24	1.8	0.60	-	-

BS1000 (1%) was added with paraquat.

Combinations of isoxaflutole and paraquat, however, were more effective than either herbicide applied alone, especially at the highest dose of isoxaflutole tested in this experiment. For example, a tank mixture of isoxaflutole 75 g a.i. ha^-1^, with paraquat 300 g a.i. ha^-1^ or paraquat 600 g a.i. ha^-1^, resulted in 83 and 92% mortality, respectively. At the highest application rates tested, the herbicide combinations did not allow the large plants of *C*. *virgata* to produce panicles (Table 5).

Compared with the control, all herbicide treatments reduced plant biomass; however, the maximum biomass reduction was achieved from the combination of isoxaflutole at the highest dose (75 g a.i. ha^-1^) and paraquat 300 or 600 g a.i. ha^-1^. The biomass reduction from the mixtures was further tested for synergy using the Colby equation [11] as previously described. As the actual values of biomass reduction from the mixtures were significantly higher than the expected values calculated using the Colby equation we conclude that this mixture is likely to be synergistic (Table 5).

### Experiment 6. Isoxaflutole and paraquat applications to roots and foliage

In the herbicide uptake experiment, an interaction was found between herbicide and method of plant uptake for both plant survival and biomass (Table 6). Plant survival and biomass reduction were similar for paraquat 300 g a.i. ha^-1^ between the foliar and foliar plus soil uptake treatments. This indicates, as expected, that root uptake is not a pathway for paraquat activity. For isoxaflutole-based treatments (37.5 to 75 g a.i. ha^-1^), and including mixtures with paraquat (300 g a.i. ha^-1^), plant mortality was greater when uptake was allowed through foliage plus soil compared with foliage alone, demonstrating that both root and foliar uptake pathways are important for this herbicide. For example, only 17% of plants survived when isoxaflutole 75 g a.i. ha^-1^ plus paraquat 300 g a.i. ha^-1^ was allowed to enter through both foliage and soil, compared to 83% survival when uptake was restricted to entry via foliage only.

**Table 6:** Effect of herbicide treatments and type of uptake on survival, panicle production, and shoot biomass of glyphosate-resistant *Chloris virgata*. Percent biomass reduction and expected Colby equation values from mixtures of isoxaflutole and paraquat were also calculated. Herbicides were sprayed at the 40–50 leaf stage.

Treatment	Survival		Panicle	Biomass		Foliar		Soil	
	Foliar	Foliar + soil		Foliar	Foliar + soil	Biomass reduction versus untreated	Expected valued (Colby equation)	Biomass reduction versus untreated	Expected valued (Colby equation)
	%	%	number plant^-1^	g plant^-1^	g plant^-1^	%	%	%	%
Control	100	100	6.3	1.97 (3.44)	1.97 (3.44)	-	-	-	-
Paraquat 300	100	100	3.8	1.40 (1.47)	1.52 (1.87)	57.3	-	45.6	-
Isoxaflutole 37.5	100	83	5.0	1.84 (3.01)	1.74 (2.61)	12.5	-	24.1	-
Isoxaflutole 75.0	100	83	4.5	1.72 (2.49)	1.49 (1.87)	27.6	-	45.6	-
Isoxaflutole 37.5 + paraquat 300	83	50	0.4	1.08 (0.67)	0.79 (0.15)	80.5	62.6	95.6	58.8
Isoxaflutole 75.0 + paraquat 300	83	17	0.2	0.99 (0.49)	0.76 (0.09)	85.8	69.1	97.4	70.4
LSD	16		0.3	0.19		-	-	-	-

Biomass data were square transformed before analysis. Original values are given in parentheses. Non-transformed biomass data was used for calculating biomass reduction and expected values.

BS1000 (1%) was added with paraquat.

Panicle number was affected by herbicide treatment (Table 6). Compared with the control, all herbicide treatments reduced panicle production; however, the maximum reduction was provided by the combinations of isoxaflutole and paraquat. Biomass was similar between the foliar and foliar plus soil treatments for control and paraquat (Table 6). Biomass was reduced when the herbicides were applied alone, however this was not statistically significant for the lowest rate of isoxaflutole when applied to foliage only. The combination treatments of isoxaflutole (37.5 or 75 g a.i. ha^-1^) and paraquat (300 g a.i. ha^-1^) provided the greatest reduction in biomass, and, when tested with the Colby equation, again demonstrated synergistic activity from this mixture (Table 6).

The results of this experiment suggest that isoxaflutole is absorbed through both the foliage and the soil (roots). This suggests that the efficacy of this herbicide combination may be reduced in a dense weed patch, where less of the isoxaflutole may reach the soil. In Australia, isoxaflutole is mainly used as a PRE herbicide in crops, such as chickpea [17]. However, it has excellent potential to be used as a POST herbicide [18]. The results of this study suggest that isoxaflutole in combination with paraquat provides additional control strategies against *C*. *virgata*. This herbicide combination appears to have potential for both reducing biomass of existing weeds, with the isoxaflutole component also providing residual soil activity that can control future emerging weeds. Such a use pattern is economically attractive [18]. Under fallow situations and potentially other non-crop areas such as roadsides and fences, a tank mix application of isoxaflutole and paraquat may provide knockdown and extended control of *C*. *virgata* if applied after the first flush of new germinations. Both herbicides have already been used in fallows to manage weeds.

In summary, GR weeds, including *C*. *virgata* have been increasing in the past few years in the eastern region of Australia which has a long history of heavy use of glyphosate in reduced tillage farming systems. Alternative herbicide options need to be identified to reduce reliance on glyphosate. Glufosinate at 750 g a.i. ha^-1^ provided high levels of control of small plants of *C*. *virgata* in these pot trails, while 1500 g a.i. ha^-1^ achieved 100% control at this growth stage. Control reduced as plant size increased; however, the highest rate tested 1500 g a.i. ha^-1^ provided promising control of larger plants.

Haloxyfop, an ACCase inhibitor, was found to be effective on both small and large plants of *C*. *virgata* and appears to be more robust than clethodim which was the other ACCase inhibitor tested in this study. Herbicides from this mode of action group, however, are prone to the risk of the development of herbicide resistance in weeds. Therefore, herbicides need to be rotated between different seasons with herbicide rates, growing conditions, and weed size carefully factored into spraying decisions to achieve high levels of efficacy. Additionally, in Australia, a sequential application of a contact herbicide with a different mode of action is recommended to be applied soon after the ACCase herbicide when these herbicides are applied in non-crop situations.

As mentioned above, post-emergent control of *C*. *virgata* may also be achieved with a combination of isoxaflutole and paraquat. While not measured in this study, the isoxaflutole component will provide additional residual soil activity. *Chloris virgata* emerges in multiple cohorts during the year. Where the preferred management strategy is to use POST herbicides, continual applications will be needed after each emergence. This both increases selection for herbicide resistance and may also result in large plants should the application be delayed e.g. wet soils or climatic conditions delaying timely POST application. Many growers managing *C*. *virgata* have turned to the use of residual herbicides to reduce the frequency of POST applications; however, no PRE herbicide provides complete prevention from emergence, and it is common to find low numbers of plants establishing after any PRE application. Often it may not be practical to control these individual plants and hence they may be large before they are controlled. For these reasons, there is a need to understand the effectiveness of alternate herbicide strategies, especially on larger *C*. *virgata*.

As mentioned above, one plant of *C*. *virgata* can produce 140,000 seeds, which can easily disperse through wind and cause infestations in paddocks, so strategies to prevent seed production are critical. Late applications of effective herbicides may be a tool to provide control or prevent seedset of *C*. *virgata*. Some treatments in these experiments did not allow the weed plants to produce seeds. If seed inputs into the seedbank are stopped, *C*. *virgata* problems in the paddock may be diminished quickly, as the seed persistence of this weed in the soil is short-lived.
